# Cryo-EM structure of *Mycobacterium tuberculosis* 50S ribosomal subunit bound with clarithromycin reveals dynamic and specific interactions with macrolides

**DOI:** 10.1080/22221751.2021.2022439

**Published:** 2022-01-21

**Authors:** Wen Zhang, ZhiFei Li, Yufan Sun, Peng Cui, Jianhua Liang, Qinghe Xing, Jing Wu, Yanhui Xu, Wenhong Zhang, Ying Zhang, Lin He, Ning Gao

**Affiliations:** a Institutes of Biomedical Sciences, Fudan University, Shanghai, People’s Republic of China; b Fudan University Pudong Medical Center, Department of Systems Biology for Medicine, School of Basic Medical Sciences, Fudan University, Shanghai, China; c State Key Laboratory of Membrane Biology, National Biomedical Imaging Center, Peking-Tsinghua Center for Life Sciences, School of Life Sciences, Peking University, Beijing, People’s Republic of China; d China National Center for Biotechnology Development, Beijing, People’s Republic of China; e Department of Medical Microbiology, Key Laboratory of Medical Molecular Virology of Ministries of Education and Health, School of Basic Medical Sciences, Fudan University, Shanghai, People’s Republic of China; f Department of Infectious Diseases, National Medical Center for Infectious Diseases, Shanghai Key Laboratory of Infectious Diseases and Biosafety Emergency Response, Huashan Hospital, Shanghai Medical College, Fudan University, Shanghai, People’s Republic of China; g School of Chemistry and Chemical Engineering, Beijing Institute of Technology, Beijing, People’s Republic of China; h State Key Laboratory for the Diagnosis and Treatment of Infectious Diseases, The First Affiliated Hospital, Zhejiang University School of Medicine, Hangzhou, People’s Republic of China; i Bio-X Institute, Key Laboratory for the Genetics of Developmental and Neuropsychiatric Disorders (Ministry of Education), Shanghai Jiao Tong University, Shanghai, People’s Republic of China

**Keywords:** *Mycobacterium tuberculosis*, 50S ribosomal subunit, clarithromycin, gate site a2062, dynamic interaction, Cryo-EM

## Abstract

Tuberculosis (TB) is the leading infectious disease caused by *Mycobacterium tuberculosis* (*Mtb*). Clarithromycin (CTY), an analog of erythromycin (ERY), is more potent against multidrug-resistance (MDR) TB. ERY and CTY were previously reported to bind to the nascent polypeptide exit tunnel (NPET) near peptidyl transferase center (PTC), but the only available CTY structure in complex with *D. radiodurans* (*Dra*) ribosome could be misinterpreted due to resolution limitation. To date, the mechanism of specificity and efficacy of CTY for *Mtb* remains elusive since the *Mtb* ribosome-CTY complex structure is still unknown. Here, we employed new sample preparation methods and solved the *Mtb* ribosome-CTY complex structure at 3.3Å with cryo-EM technique, where the crucial gate site A2062 (*E. coli* numbering) is located at the CTY binding site within NPET. Two alternative conformations of A2062, a novel *syn*-conformation as well as a swayed conformation bound with water molecule at interface, may play a role in coordinating the binding of specific drug molecules. The previously overlooked C–H hydrogen bond (H-bond) and π interaction may collectively contribute to the enhanced binding affinity. Together, our structure data provide a structural basis for the dynamic binding as well as the specificity of CTY and explain of how a single methyl group in CTY improves its potency, which provides new evidence to reveal previously unclear mechanism of translational modulation for future drug design and anti-TB therapy. Furthermore, our sample preparation method may facilitate drug discovery based on the complexes with low water solubility drugs by cryo-EM technique.

## Introduction

TB is the leading infectious killer ranking above HIV/AIDS and malaria. The increasing emergence of drug-resistant TB worldwide, calls for urgent development of new drugs [1]. Ribosome is a major target for half of all antibiotics, including macrolides ERY and its derivative CTY [2].

**Figure 1.**
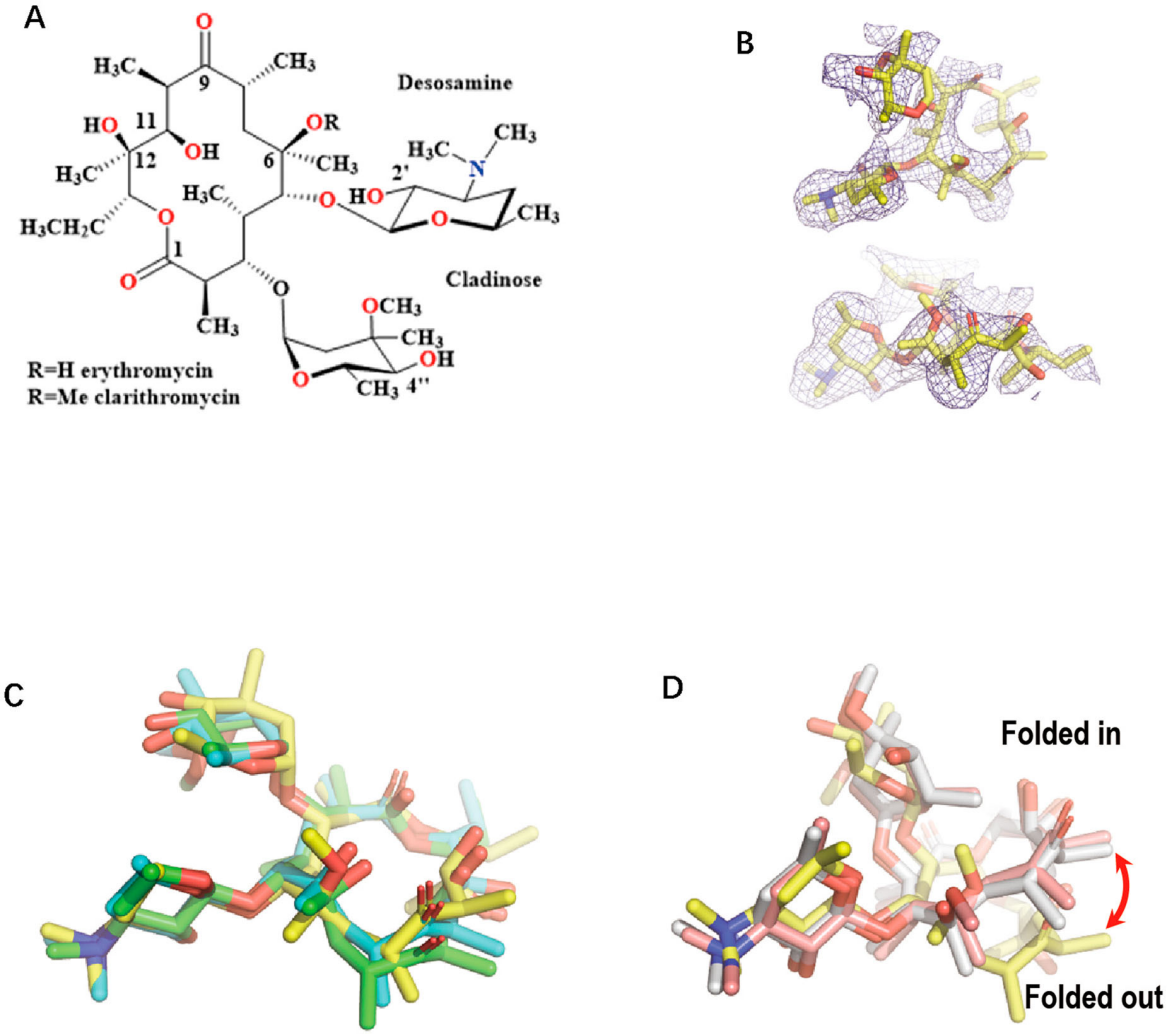
The comparison of the *Mtb* ribosome-bound clarithromycin (CTY) with the antibiotics from different ribosome complexes. (A) Chemical structures of NPET binding Erythromycin (ERY) and Clarithromycin; (B) The density map (deep blue mesh) of CTY in complex with the *Mtb* 50S ribosome viewed from two perspectives (maps contoured at 1.7σ and 1.2σ, respectively); (C) Comparison of the ribosome structures in complex with ERY or CTY (CTY carbon in yellow, PDB entry 7F0D, 50S of *Mtb*), ERY (carbon in green, PDB entry 4V7U, 70S of *E. coli*; carbon in cyan, PDB entry 6XHX, 70S of *T. thermophilus*) (D) Comparison of the 50S ribosome structures in complex with ERY or CTY (carbon in yellow, PDB entry 7F0D, *Mtb*), CTY (carbon in light pink, PDB entry 1J5A, *D radiodurans*) and ERY (carbon in grey, PDB entry 1JZY, *D radiodurans*).

CTY is a second-generation macrolide that is widely used to treat respiratory infections and has been used as an anti-TB agent. Although it has a modest bacteriostatic activity, CTY has been reported to augment the activities of many other anti-TB drugs, with good tolerability and safety profile, such as prolonged treatment of MDR-TB in combination with four other anti-TB agents [3–5]. In addition, CTY is a frontline drug used for the treatment of nontuberculous mycobacteria (NTM) infections [6]. For example, CTY is highly potent in treating diseases caused by *M. avium*-intracellulare complex strains, with the MIC being 4 μg/ml, compared to ERY with 64 μg/ml [7]. Therefore, CTY is a potent anti-mycobacterial agent that should be further investigated and evaluated [3]. However, it is still unclear why CTY is more active than ERY, despite the two antibiotics having only a methyl group difference [8,9] Figure 1(a). The structural basis for the differential activity of CTY and ERY for *Mtb* has been lacking [10,11].

Most of the deposited complex structures of macrolides and bacterial ribosomes are resolved from Archaea and *E. coli* rather than clinically relevant pathogens [2]. There are species-specific features for some antibiotics like telithromycin (TEL), but there is little structural information to reveal the mechanism of drug specificity for pathogens [12–14]. Thus far, the only one available ribosome-CTY structure from *Dra* was solved at 3.5 Å due to the challenge of low water solubility of CTY [15]. The previously reported structures of CTY and ERY bound to *Dra* ribosome is attributed to erroneous density map interpretation related to the resolution limit [14,16–18].

It is generally believed that NPET is inert. A2062 is one of key nucleotides to interact with CTY within NPET, and it is the closest nucleotide interacting with the 6-hydroxy group of ERY. Particularly, the dynamic of A2062 is related to gating the nascent peptide chain translation, which is crucial for selective translation and translational modulation [19]. How the A2062 gate plays a role in the CTY binding and contributes to specificity has been elusive.

Thus, it is worthwhile to elucidate the detailed structural basis for the interactions between CTY and the *Mtb* 50S ribosome. We previously found that it was difficult to get a good structure or clear density map of CTY moiety even though we tried long time incubation with the ribosome in a saturating concentration of CTY, due to the very low solubility of CTY (0.33 mg/L) when forming ribosome-CTY complex for cryo-EM sample. Moreover, although the additional organic solvents may help to increase the CTY solubility, the high concentration organic solvents may result in ribosome damage or high noise background in cryo-EM images. For the same reason, it is challenging to obtain any antibiotic density from the cryo-EM ribosome structure in complex with linezolid (LZD) due to its low solubility (about 2 g/L). Thus, the lack of structural interaction insights of the ribosome-antibiotic complexes at high resolution undermines the efforts at rational design of novel chemicals against drug-resistant TB.

The high-resolution ribosome structure from *M smegmatis* (*Msm*) and *Mtb* has been solved recently at 3–4 Å [20–23]. However, few ribosome-antibiotics complexes have been reported except a linezolid analog (LZD-114) with 20 times more potency than linezolid [21]. In previous reports, the conformational change of A2062 may represent the specific response to the binding of a macrolide antibiotic. The ERY-induced reorientation of A2062 is observed only in some species like *T thermophiles* (*Tth*), but not *E. coli* (*Eco*), *H marismortui* (*Hma*) and *S aureus* (*Sau*)[24,25]. Curiously, the mechanism of the species-specific and macrolide-specific dynamics of A2062 remains unclear since many solution structures of ribosome-antibiotics at high resolution are still unavailable.

In this study, we used a novel buffer exchange method to prepare the ribosome-CTY complex, which was frequently used in soaking crystals [26]. After many trials and optimizations, we have solved a cryo-EM complex structure of *Mtb* 50S and CTY at 3.3 Å, with the CTY map intensity better than those from the conventional soaking method. The higher resolution electron density map, local resolution better than 3 Å, within the NPET allowed us to place most atoms of CTY at the interface, and to reveal the specific dynamics of A2062 in the ribosome-CTY complex. The structure insights from this work not only demonstrate the interaction details of the ribosome-CTY complex but also provide insights on how anti-TB potency of CTY is improved by introduction of a single methyl group.

## Materials and methods

### Ribosome purification

1. M. tuberculosis H37Ra avirulent strain was cultured in Middlebrook 7H9 liquid medium with 0.2% Glycerol, 0.05% Tween-80 and 1.5% glycine at 37°C until OD600 –1.0. Cell was harvested at 4°C and cell pellets were lysed in bead beater (Biospec) with buffer A (20 mM Tris-HCl at PH 7.5, 100 mM NH4Cl, 20 mM MgCl2, 5 mM 2-Mercaptoethanol, 0.5 mM EDTA). The Bead beater is covered with an additional plastic envelope, and all operations are performed in the Biosafety cabinet. The cell lysate was centrifuged at 18,000 rpm for 100 min. The clear supernatant was loaded on a sucrose cushion in buffer BS (20 mM Tris-HCl-pH 7.5, 500 mM NH4Cl, 10 mM MgCl2, 5 mM 2-Mercaptoethanol, 0.5 mM EDTA, 1.1 M Sucrose), and spun at 28,000 rpm in a Ti45 rotor for 16 h. The crude ribosome pellets were resuspended in buffer A and applied the Source-Q column (GE Healthcare) with eluting ionic gradient from 60 mM to 1M NH4Cl in the Buffer A. The ribosome peaks were changed to the buffer A before loading to the 15%–45% sucrose gradient and then spun down in Beckman SW32 rotor at 28,000 rpm for 16 h. The ribosome peaks collected from the 70S and 50S fractions were changed to buffer A and frozen at −80°C for future use [22].

### Sample preparation and data collection

With the modified protocols from crystal soaking with antibiotics, we performed the antibiotics binding reaction for the cryo-EM samples [26]. During thawing out the ribosome, CTY(Sigma-Aldrich) in stock solution (50 mM stock with acetone) was added at a 4% stock solution (CTY concentration at 2 mM) to the ribosome sample, and the mixture solution was incubated at 37° with CTY containing buffer A. After 30 min of incubation, the ribosome sample with CTY stock solution was applied (CTY at 100 μM or stock solution at 0.2%) by buffer exchanging with Buffer A and spinning down with Millipore Amicon Ultra-0.5 Centrifugal Filter Unit.

4-µL aliquots of purified ribosomes at a concentration of 100 nM were applied to carbon-coated grids (Quantifoil, R2/2). Grids were blotted with filter paper at both sides for 2 s using FEI vitrobot Mark IV (100% humidity, 4°C) and vitrified in liquid ethane. The cryo-grids were transferred to a transmission electron microscope (FEI Titan Krios) operating at 300 kV for data collection. Images were recorded, at a nominal magnification of 18,000 X, on the Gatan K2 Summit detector in counting mode. Under these conditions, the pixel size at the object scale is 1.04 Å. The nominal defocus used to collect data ranged from −1.5 to −2.5 µm. All image stacks were collected with Gatan Digital Micrograph at low-dose conditions with each stack containing 32 dose-fractionated frames. The total exposure time for each stack was 8s and the dose rate for each pixel was ∼8 counts [22].

### Image processing, model building and refinement

The stack frames were aligned to create motion-corrected micrographs by MOTIONCORR [27], and the contrast transfer function (CTF) parameters were estimated by CTFFIND3 [28]. The particles were classified in 2D and 3D steps by RELION [29] after the initial picking by SPIDER. Several rounds of 3D classification were carried out to improve the resolution of the density map, and six similar classes were combined for the following rounds of 3D classification. Only one class of 50S was selected for the high-resolution refinement (34,912 particles). The final refinement for the 50S were performed with soft-masks, together with the dose-reduced micrographs (particle set selected from frame 2 to 15 by MOTIONCORR) [22]. The final resolution for the *Mtb* 50S is 3.31 Å (RELION 3 with criterion: FSC = 0.143).

The previous *Mtb* 50S structure (PDB: 5V7Q) was fitted in the *Mtb* 50S map. The protein and RNA fragments with very weak density were deleted, and the fragments with large shifts were manually adjusted in Coot [30]. The CTY model was extracted from *Dra* ribosome-CTY complex (PDB: 1J5A), and manually fit and adjusted into the density map. The alternative structure model of A2062 was adjusted in Coot and manually modified in the structure model. The real-space refinement was performed for the *Mtb* 50S-CTY model against the 3.31 Å cryo-EM map by PHENIX [31]. Additional manual adjustments were applied in Coot after the refinement. The final model was evaluated by Molprobity [32] after several rounds of refinement and adjustment. The structure of CTY remained a negligibly small change after structure refinement against dual conformations of A2062 or *syn-*A2062, so the same CTY structure was chosen for both conformations.

The structure alignments were carried out with command align in Pymol. The further alignment against the domain V of 23S rRNA was used to compare the structures of antibiotics and nucleotides within NPET. The same translation matrix of 50S was chosen for the alignment of tRNA and other structures in 30S when the 70S ribosome structure was compared with the *Mtb* 50S ribosome.

π-interaction and C–H H-bond were displayed by Discovery studio (BIOVIA, Dassault Systèmes) or MOE (Molecular Operating Environment). The report of the interactions between ribosome and CTY was also created by Discovery studio or MOE, and the 2D plot of interaction was generated by Discovery studio.

### Accession codes

The cryo-EM density map of the *Mtb* 50S has been deposited in the EMDB with accession number EMD-31398 The atomic model has been deposited in the PDB with accession number 7F0D.

## Results

Here we report the structure of *Mtb* 50S ribosome-CTY complex at 3.3 Å. The detailed molecular interactions, dynamic behaviour and functional implications will be discussed below.

### CTY bound to *Mtb* ribosome is consistent with other ribosome-ERY complex

**Figure 2.**
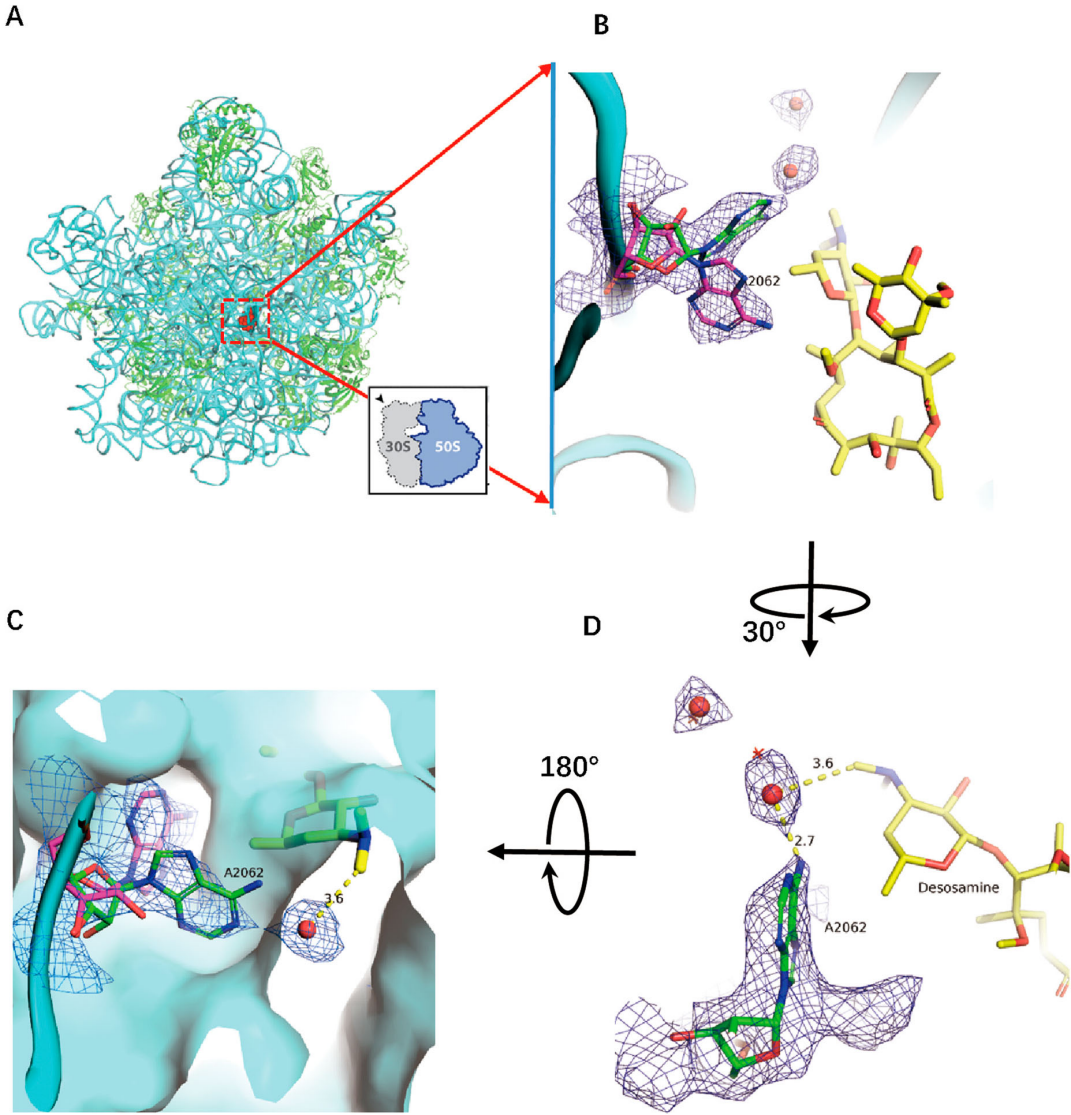
Structure of CTY in complex with the *Mtb* 50S ribosome and the close-up view. (A) Overview of the CTY binding site (red in square) in the *Mtb* 50S ribosome, view from the 30S side as indicated by the inset; Close-up views of CTY bound within the NEPT, together with two alternative conformations of A2062, non-swayed conformation in purple carbon and the swayed conformation of A2062 in green carbon (B) and (C); (D) The revised view with the surface (in cyan) for the macrolides binding site. Notes: The density maps for A2062 and nearby water molecules are contoured at 1.8 in mesh; The *E. c*oli nucleotide numbering is used; the distances between water molecule and nearby atoms are shown in yellow dashed lines. The water molecules are in small red spheres from Mtb structure compared with the corresponding waters in wheat spheres from two structures (PDB codes 6XHX and 6XHY).

As the second-generation derivatives of ERY, CTY with a methoxy group substituted on the 6-OH, has improved acid stability and activity (Figure 1(a)) [8,9,33]. The previous ribosome-ERY structures were superposed to *Mtb* 50S-CTY structure, and the antibiotics were further compared by central density map fitting. In our structure, the CTY molecule is located in the canonical macrolide binding site within NPET, which is similar to ERY bound to 70S ribosomes from *Eco* and *Tth* (Figures 1 and 2) [16,25,34].

The overall structure of CTY bound to *Mtb* 50S ribosome is similar to the ERY structures in complex with ribosomes from other species, except there is a large deviation of the lactone ring compared to the structures of CTY and ERY bound to *Dra* 50S ribosome, suggesting incorrect folded-in conformation mainly due to the misinterpretation for limited resolution map near the binding site at 3.5 Å (Figure 1(d)) [14,17]. Our CTY structure solved at 3.3 Å, with better local resolution (3 Å or better) around the macrolide binding site, is identical with the folded-out conformation from the most ribosome-ERY complexes (Figure 1(c)).

From the solution structure of cryo-EM map, the structure dynamics and binding affinity could be revealed from the local density map. The electron density is strong around desosamine moiety of CTY, but relatively weak around the opposite side of the lactone ring of CTY, which has a similar density pattern in the 2.4 Å cryo-EM map of the ribosome-ERY complex from *S aureus* (*Sau*) [24].

The desosamine is favoured for tight binding in most macrolide antibiotics, while the part of the lactone ring is relatively flexible, which could be stabilized by the extra branch of antibiotics like TEL. The density of cladinose linker had weak intensity due to the possible dynamic conformation, especially as it can contact nascent peptide chain (NPC) during translation.

### Alternative conformation of A2062 is observed in 50S-CTY complex.

CTY is bound to the site in NPET close to the P-site, which includes the nucleotides 2503, 2505, 2508, 2509 and A2062 (*Eco* numbering, Supplementary Table 1). A2062, so called the gate site, is one of the key residues to interact with CTY, which is crucial in protein translation control [19]. The preview reports show A2062 adopt either unrotated conformation in ribosome from *Eco*, *Sau* and *Hma* or rotated conformation in *Tth* ribosome [25]. In our structure, there are dual conformations observed from the density map (the close-up view from the 30S side (Figure 2(b)), which are located on the two sides of the 5'-methyl group of desosamine. Besides the similar density of non-swayed conformation, the swayed A2062 closely contacts a putative water molecule with a distance around 3 Å to the A2062 and desasomine (Figure 2(c)). Most likely, there is a H-bond between the water molecule and the N6 atom of A2062. These two water molecules near desosamine are very close to the corresponding water molecules observed in the previous structures (PDB codes: 6XHX, 6XHY, 6XHV, 1YHQ; the position deviation of water molecules from these compared structures is around 1 Å), thus they may have the similar function due to their close positions(Figure 2, S3, S5A) [34]. From the surface map of the binding site, the water bound to A2062 fill the surface gap near desosamine, which may involve stabilization of CTY binding (Figure 2).

### The structure comparison of A2062 conformations from different complexes

**Figure 3.**
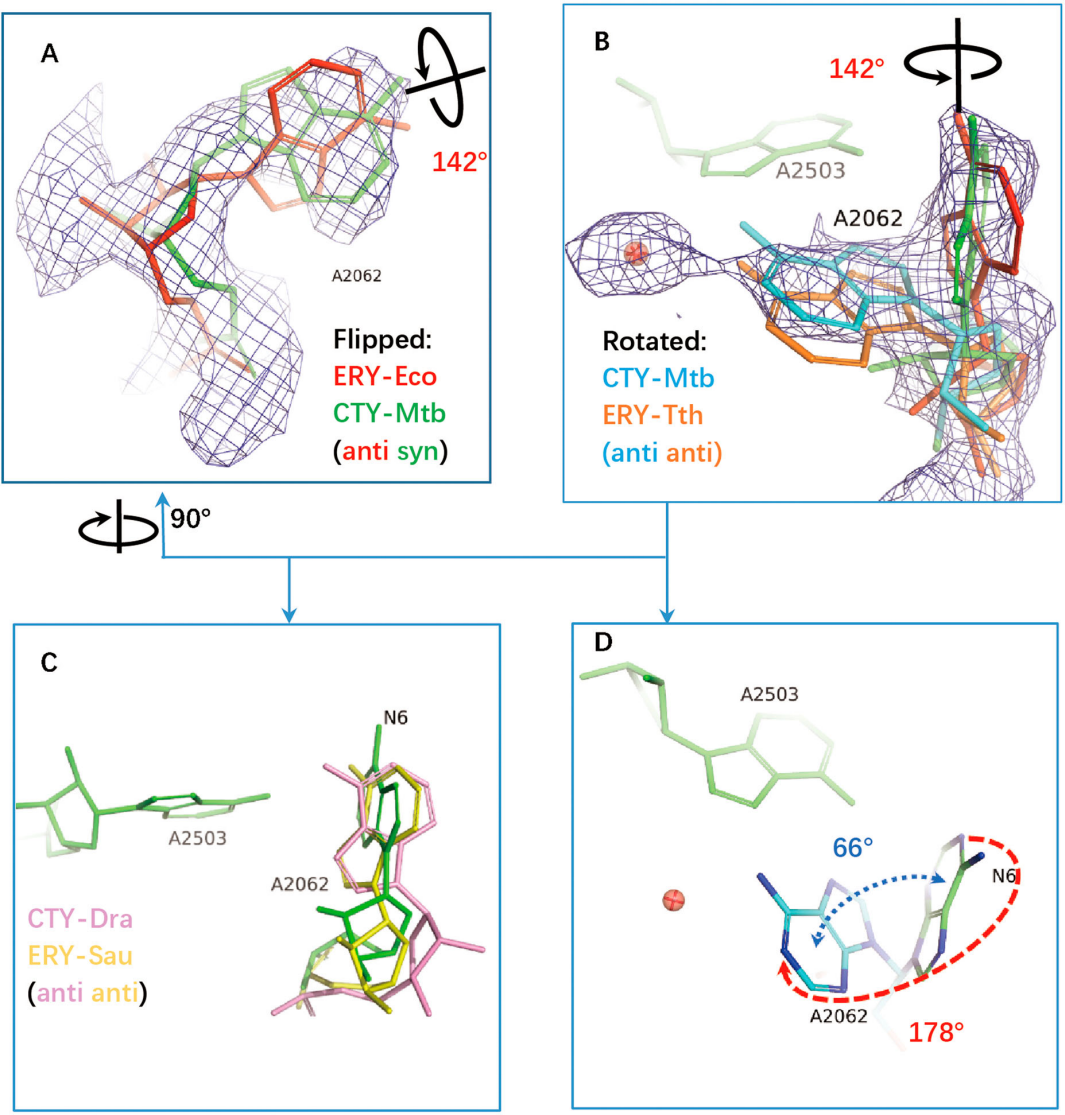
The structure comparison of A2062 in the ribosome complexes with CTY or ERY. (A)(B) The alternative conformations of A2062 with density map contoured at 1.5 from different views (syn-A2062 in *Mtb* 50S ribosome-CTY complex or CTY-*Mtb* ribosome complex in green, anti-A2062 in ERY-*Eco* complex from PDB 4V7U in red; A2062 in ERY-*Tth* ribosome complex from PDB 6XHX in orange, with 1 Å deviation of base comparing to the anti-A2062 in CTY-*Mtb* ribosome complex in cyan); (C) The A2062 structure comparison between the non-swayed conformations from different ribosomes (CTY-Dra from PDB 1J5A in pink, ERY-*Asu* from PDB 6S0X in yellow); (D) The A2062 rotation between two conformations (the rotation about 178°, while the plane angle of two bases is about 66°).

The structures of antibiotics in complex with ribosomes from *Eco*, *Tth*, *Dra* are compared by superposing to *Mtb* 50S ribosome structure and optimized by docking the structures into the *Mtb* density map around the binding site within NPET. The specific *syn* conformation of A2062 (*syn-*A2062) identified in 50S-CTY of *Mtb* is about 142° base rotation comparing to the *anti-*conformation of the canonical unrotated A2062 in the *Eco* ribosome-ERY complex (Figure 3(a, b)).

Further comparing A2062 conformation of the *Eco* ribosome-ERY complex with vacant ribosomes from *Mtb*, *Msm* and *Eco* show the later conformations are variable since their conformations are flexible, together with the weak density (Figure S4). The remodelled A2062 of the vacant *Mtb* ribosome at low resolution show 45° rotation compared to our 50S-CTY structure, while the observed density in our structure is clear enough to show the stable binding between A2062 and CTY (Figure 2 and 3, S4).

**Figure 4.**
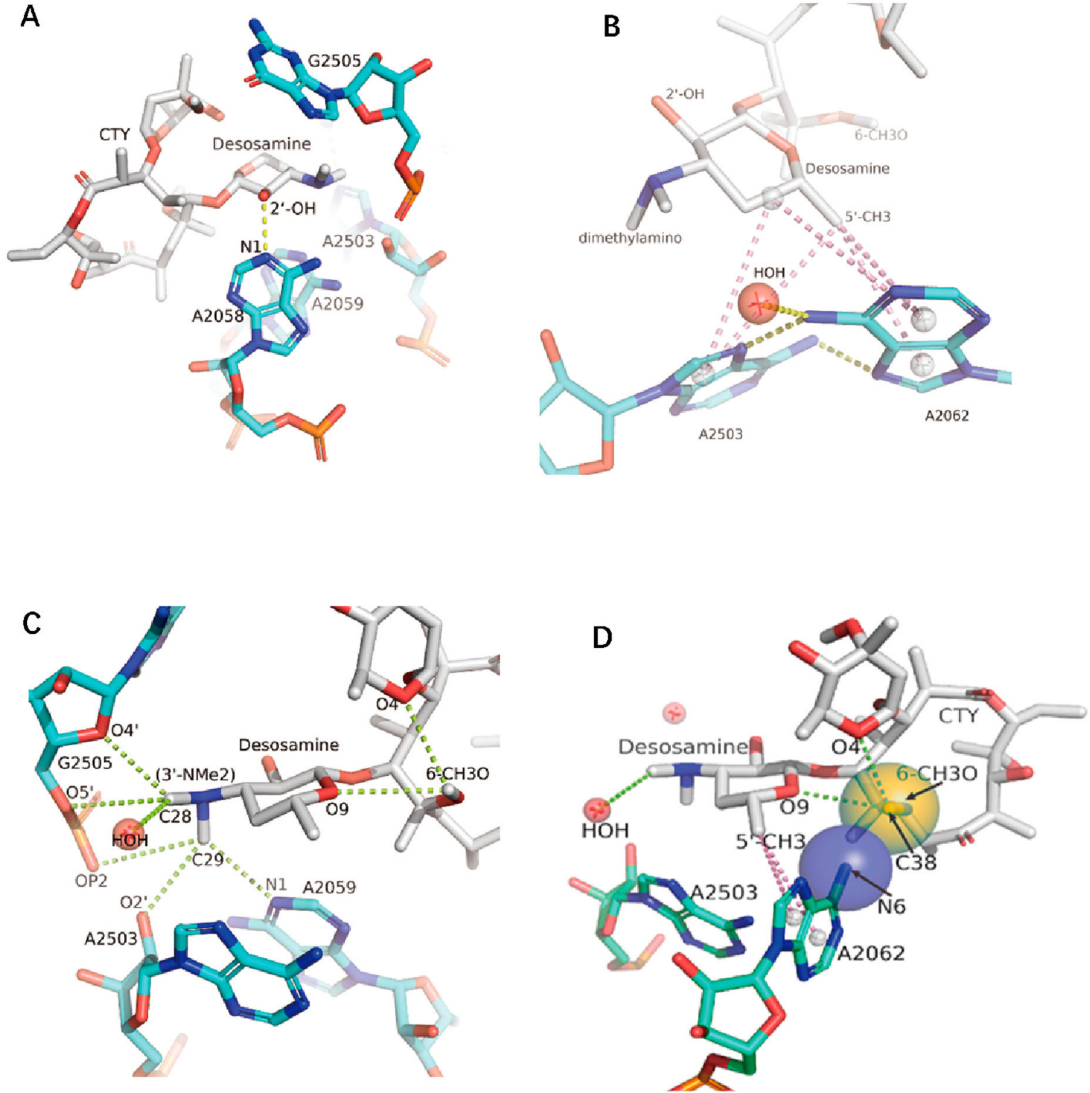
The interactions of CTY with the ribosome. (A)The major nucleotides of the NEPT interacting with CTY from A2058 side view; (B) The π-interactions between desosamine and a Hoogsteen base pair formed by m^2^A2503 and the swayed A2062; (C) The interactions between Dimethylamino group (3’-NMe2) of desosamine and m^2^A2503, G2505 and A2059; (D) The interactions of the N6-methoxy Bridge between CTY and the non-swayed A2062 (N6 of A2062 in blue sphere, methylation group of 6-methoxy of CTY in orange sphere); Notes: H-bonds in yellow dotted lines, π-interactions in pink dotted lines, C–H H-bonds in limon dotted lines.

Moreover, the swayed A2062 in *Tth* ribosome-ERY complex (PDB code 6XHX) is about 1 Å deviation compared to our structure, mainly due to the variation from structure superposition, Figure 3(b). Therefore, the swayed conformation of A2062 in our structure is consistent with the rotated A2062 reported previously [25,34], which leads to forming the Hoogsteen base pair with m^2^A2503 (Figure 4(b), S1, S2).

The 178° base rotation of A2062 between two conformations in our structure is close to the canonical rotation in *Tth* ribosome-ERY complex (about 160°), while the base plane is only swayed about 66° against the *syn* conformation in our structure.

The base plane of A2062 in *Dra* 50S-CTY is almost vertical with our structure and has a large deviation compared to the plane of ribosome-ERY structures from *Sau* and *Eco*, which may result from the incorrect folded-in conformation of CTY interpreted at a limited resolution map (Figure 1(d)).

### The interactions of CTY with *Mtb* ribosome and resistance mechanism

The binding site for CTY is almost identical for ERY due to the highly conserved local rRNA structure within NPET. The major interactions between CTY and ribosome NPET include the H-bonds and van der Waals (VDW) contacts. The H-bond between A2058 and 2′-OH of the desosamine sugar is observed, together with the tight vdW contacts with several key nucleotides (2062, 2503, 2505, 2058) around desosamine (Figure 4, Figures S1C, D, S2 and Table S1). The swayed conformation of A2062 forms a Hoogsteen base pair with m^2^A2503 and possible base stacking with G2061 (Figure 4(b); Figure S2C).

Besides the Watson–Crick pairs, early studies showed the C–H H-bonds could not be dismissed due to their frequent observation in RNA structure and their surprising stability[35]; and recent studies identified two types of π interactions in nature from over 1000 instances of water-nucleobase stacking contacts in a variety of RNA molecules [36]. The C–H H-bonds could even be measured by atomic force microscopy although it’s relatively weak [37]. We further analyzed the collective effects of these weak bonds in the ribosome-antibiotics complexes.

Notably, there are sigma-π, alkyl-π interactions as well as the C–H H-bonds between CTY and nucleotides in NPET, which are identified and displayed by Discovery studio or MOE (Figure 4(c), S2). The total five π-interactions are involved in the interface between the desosamine sugar and the A2062/A2503 bases, and five possible C–H H-bonds between the dimethylamine of desosamine and A2503/G2505/A2059 bases with fairly conserved conformation could form a complex network of H-bonds. The dimethylamine with a relatively high pKa tends to be protonated under weakly acidic conditions contributed by nearby phosphate and negatively charged groups of ribose. The positive charge on the dimethylamine would potentially help stabilize the C–H H-bonds in the binding pocket due to Coulombic contributions. These similar weak interactions are commonly observed in the structures of ribosome-bound macrolides from other species.

The total contribution of these interactions at dimethylamine moiety may be equivalent to a canonical H-bond according to calculations (Supplementary Table S2). Therefore, C–H H-bonds are no longer ignored, although each of them is relatively weak, which is also noted by the previous studies [35,37]. The π-interactions may contribute to the major part of vdW interactions and enhance the affinity at some moieties [36].

Four π interactions involve the specific binding of A2058 with CTY, together with a canonical H-bond with 2′-OH of CTY (Figure 4(a), S2a). The mutation A2058U/C would destroy the above interaction and reduce the binding affinity, which leads to drug resistance for macrolide antibiotics [34,38]. Similarly, the drug resistance of A2059C may result from the breaking of the π interactions and C–H H-bond with macrolide (Figure 4(a), S2) [38].

The *syn-*A2062 mainly interacts with the CTY by vdW contacts. The 6-methoxy group of CTY has the VDW contacts with A2062 as well as two possible intramolecular C–H H-bonds with the desosamine and cladinose, which could stabilize the CTY structure and reduce the repulsion between negatively charged oxygen atoms (Figure 4(c,d)). The electrostatic interaction is also involved between N6 of A2062 and negative charged groups of CTY (6-methoxy group and oxygen atoms from the cladinose, with a distance around 4.2–4.5 Å) together with the VDW contact, which make the interactions more stable (Figure 4(d), S1C). The *syn-*A2062 also interacts with desosamine by π-interactions to stabilize its conformation (Figure 4(d), S2B). Therefore, the interaction related to N6 of *syn-*A2062 and the 6-methoxy group of CTY (the N6-methoxy Bridge) is crucial to contribute to the binding affinity and dynamics, which is related to CTY a broader drug spectrum of activity contributed by the novel alternative conformation.

The Interactions with both conformations of A2062 for CTY binding within the NPET site are crucial. Therefore, the mutants, A2062G and A2062U may disrupt the 2503/2062 base pair or the N6-methoxy Bridge and lead to reducing the affinity and dynamics significantly, which results in drug resistance as well as changing the spectra of ERY-resistant proteins [19,39].

### The A2062 conformation dynamics and its nearby water molecule interaction

So far, the rotated or swayed A2062 is only found from *Tth* ribosome-ERY complex and *Mtb* ribosome-CTY complex. It remains curious how the reorientation of A2062 could represent the species-specific and macrolide-specific response to the antibiotic binding [25]. Several structures around 2–3 Å were compared around A2062 to gain insight into the structure and mechanism.

The swayed A2062 in *Mtb* ribosome compared to chloramphenicol (CHL) in *Tth* 70S-CHL complex (PDB code: 6ND5), where the dichloroacetic moiety of CHL forms H-bond with the N6 atom of A2062 and induces the A2062 rotation [25] (Figure S1A, B). The water molecule bound with the swayed A2062 in *Mtb* ribosome is very close to the oxygen atom of the dichloroacetic group and forms the similar H-bond with A2062 (Figure S1A). Therefore, this water molecule can be regarded as a water pharmacophore (WP), and provide a stable interface of macrolide binding sites. However, the corresponding water molecules near WP in other structures (PDB codes: 6XHY, 6XHV and 1YHQ) are about 1 Å away from the water molecule in our structure, and could stabilize macrolide by forming a possible C–H H-bond with the desosamine dimethylamino (Figure S3, S5A).

The water molecules are remodelled around the unrotated A2062 from the ribosome in complex with Azithromycin (ZIT, another ERY analogy, PDB code: 1YHQ) (Figure S3). There are π interactions between A2062 and two water molecules, while one of them is identical with the water molecule in the vacant ribosome from *E. coli* at 2.0 Å resolution (PDB code 7K00), and another has possible H-bond with neighbour nucleotide A2061, Figure S3. Furthermore, a H-bonds of A2062 with m^2^A2053 and two additional water molecules, together with the interactions with ZIT may further stabilize the A2062 conformation, Figure S3.

In addition, comparing our structure to the ribosome with aminoacylated tRNAs (PDB code 6XHV), the swayed A2062 and the bound water molecule are close to the aminoacylated tRNAs at A-site and P-site around vdW distance (Figure S5A). Furthermore, A2062 adopts the rotated but non-swayed confirmation when the ErmCL nascent chain is bound to *E. coli* 50S-ERY complex (PDB code 3J5L; Figure S5B), where the unrotated A2062 would conflict with the ErmCL nascent chain (PDB code 3J7Z; Figure S5C, D). Therefore, it is interesting to suggest that the water molecule bound with A2062 may also involve the prepeptide-bond formation and the early stage of NPC release.

The early studies shown m^2^A2503 may involve possible *syn-anti-*conformation transition when the specific nascent peptide is present in the NPET, and mutations of residues A2062/A2503 prevent stalling [40,41]. Our structure implies that the dynamic A2062 may also involve conformation transition during CTY binding, and the water molecule binding may play a role to modulate A2062 dynamics during the dynamic translation and antibiotic translation arrest.

Mutants A2062G/A2062U may lose the H-bond with the water molecule as well as disrupt the Hoogsteen base pair with m^2^A2503, which is consistent with the early findings [19,40,41]**.**

### A2062 dynamics associated with specific macrolide binding may modulate the peptidyl transferase center (PTC) towards translation arrest

To study the dynamic and specific interactions with macrolide binding, we carefully checked and examined the previously published structures of the ribosomes in complex with Solithromycin (SOL), Erythromycin (ERY), Telithromycin (TEL) and Cethromycin (CET) [42–44].

The density for alternative conformations of A2062 in the ribosome-SOL complex and ribosome-TEL complexes could also be found similar to the conformations in the ribosome-CTY complex, except the non-swayed A2062 remains *anti-*conformation with various populations, Figure S6, S7.

The major population of A2062 prefers non-swayed conformation in the vacant ribosome-TEL complex, but tends to rotate about 90° upon the nascent polypeptide chain (NPC or ErmDL) binding, while A2062 remains non-swayed conformation in ERY binding form even the ErmDL is present. The minor alternative conformation exists in both ribosome-TEL structure forms [44], Figure S7.

Based on the ribosomes in complex with SOL, ERY and CET, the detailed molecular dynamics simulation reported the distribution of torsion angles of A2062, which clearly displayed two peaks of frequencies corresponding to the two conformations of A2062 [43]. The angle-varying frequencies are sensitive to the macrolide types. Consistently, the swayed alternative conformation is relatively weak and belongs to the minor population in the ribosome-SOL complex, Figure S6.

The interface between A2062 and macrolides indicates the interactions of A2062 with ERY are stronger than interactions with SOL and CET, which is also observed in the differences in terms of the vdW contacts, HB bonds and free energy calculations [43], Figure S3, S6B. With the 6-methoxy macrolide introduced at the interface, A2062 may become more flexible than the ERY bound form, Figure 4(d), S6B.

In previous studies, macrolides can allosterically alter the dynamics of U2585 in PTC and thereby result in the ribosome translation arrest for some specific sequences, which is revealed by biochemical probing and molecular dynamics simulations even for the binding of a macrolide to the vacant ribosome [42,45]. With the macrolide binding, the A2062 rotation could trigger a dynamic path to change the orientation of U2585 in the peptidyl transferase center (PTC).

The additional simulation and experiments show that the swayed conformation of A2062 in ErmDL-TEL-stalled ribosomal complex (SRC) is also correlated to the induced state of U2585, but no induced state of U2585 is found in the ERY bound form with the unrotated A2062 [44]. In the case of ERY binding with A2062, the ribosomal base A2062 has a high probability of contact with ERY in simulation, but the strong interaction and stable conformation may restrict its flexibility and dynamics, which indicates the important role in inhibiting protein synthesis [43]. Kannan et al. showed that the mutation A2062U confers resistance to ERY derivatives [46], instead of ketolide (TEL/SOL). The above discoveries may imply that translation modulation may be influenced by the affinity advances of alkyl-aryl side chain as well as the dynamic contributions of 6-methoxy of TEL [44].

Besides the A2062 dynamics exhibiting species-specific features, the A2062 prefers alternative conformations for specific binding and dynamic regulation when 6-methoxy is presented in macrolide, which may provide a key mechanism for CTY with superior potency compared to ERY, although some macrolides have a similar binding affinity according to the recent study [43]. Besides CTY, the macrolide with 6-methoxy, like TEL and SOL, may have gained extra potency for synergistic effect through the A2062 dynamics regulation. This specific feature can be applied in drug screening with various modifications at 6-OH for further drug design.

## Discussion

A better understanding of the structure activity relationship for anti-TB drugs is urgently required to investigate the promising TB therapy as well as the structure-based drug design. For some reasons, ribosomes of the pathogens especially the *Mtb* ribosome in complex with antibiotics are less accessible to medicinal chemists, which makes the rational drug design more difficult. In this study, we determined the cryo-EM structure of *Mtb* 50S ribosome-CTY complex which provides the dynamic interactions of the antibiotic CTY within NEPT and reveals the detailed mechanism of translation modulation and drug resistance of *Mtb* ribosome. Particularly, the crucial gate site, A2062, provides two binding conformations for CTY to modulate translation and translational control. More importantly, our current work reports a new structural finding that would be relevant to other ribosome-targeting anti-TB drugs, such as LZD and for virtual screen to identify potential new inhibitors targeting the TB 50S ribosome. Furthermore, the TB ribosome structure we solved here should serve as a model for understanding how macrolides bind to corresponding ribosomal sites in nontuberculous mycobacteria (NTM) where macrolides are drug of first choice for treatment [6].

There has been a longtime debate on the lack of antibiotics structure consensus from different species in two decades, although many ribosome-antibiotics structures have been solved since 2001 [15,18,25]. Two studies of erythromycin (ERY) and related macrolides interactions were revisited on ribosome from *Thermus* and *E. coli* in 2010 [14,16] and vindicated the folded-out conformation for the macrolide lactone ring compared to the early folded-in conformation from *Deinococcus* in 2001 [15]. The discrepancies from the early structure data could be caused by problems with the limited resolution and interpretation of the data other than the species differences in the ribosomes [18]. Therefore, antibiotics structures from pathogens are indispensable to delve deeper into the atomic details at higher resolution [18,47].

The Mtb ribosome-CTY structure near 3 Å we solved here are in agreement with the lactone ring conformations of ribosome-ERY structures from *Thermus, E. coli and S aureus* as well as the folded-out conformation for *Dra* 50S-ERY structure revised from early folded-in conformation of the lactone ring (the results from the unpublished structure data in 2005; Figure 1, S2) [14,16–18].

Notably, the strong map intensity of the swayed A2062 and nearby water molecule is correlated to the major population of ribosome-CTY complex, while the relative weak density of the *syn-*A2062 indicates the minor population of A2062 conformation. The density map is to some extent related to the binding affinity and dynamics. The strong density around desosamine moiety is mainly contributed by many favourite H-bonds and π interactions (Figure 4), while the weak density around the opposite side lactone ring is related to the unfavourite interaction with U2611 (Figure 1(b), S2B, D). These findings are consistent with the facts in drug design that the desosamine of ERY derivatives is rarely changed but an extra branch of macrolides like TEL or solithromycin (SOL) is introduced to strengthen antibiotics binding and potency.

The water molecule near WP bound to the swayed A2062 may provide an additional interface for antibiotic binding, and it is close enough to the swayed A2062 to form H-bond found in our structure. The structure dynamics of A2062 revealed by dual conformations may imply the specific dynamic process for both *syn-anti* and sway transition when A2062 adopting new conformations to respond the specific binding involved in methoxy group of CTY at interface, which is similar to the A1493 adopting novel *syn* conformation to fit the unusual base pairing during the decoding of a pseudouridylated stop codon by the ribosome [48]. Alternatively, this water molecule near WP could be identified to form C–H H-bond with dimethylamino by software due to the possible position within contact distance, and eventually it could potentially bridge the water-mediated interactions between A2062 and dimethylamino (Figure 2, S2, S3) [35,37].

The swayed A2062 forming the Hoogsteen base pair with m^2^A2503 as well as the H-bond with nearby water molecule leads to the stable binding site and the tight vdW contacts enhanced by π-interactions, together with the possible C–H H-bonds between CTY and NPET nucleotides (A2503, G2505, A2059). The C–H H-bonds and π interactions are relatively weak, but may collectively play a role in the ribosome-antibiotics interactions, which should be taken into account during the structure analysis and drug discovery [35–37]. Meanwhile, the N6-methoxy Bridge with *syn-*A2062 mediates the favourable interactions in terms of the antibiotics internal stabilization, a better fit for the binding site and the possible pivot for A2062 rotation, which may significantly contribute to the binding affinity as well as the dynamic interactions. Altogether, the gate site A2062 adopts alternative *syn* conformations to interact with CTY tightly, in addition, the 6-methoxy group of CTY and water molecule interacting with the corresponding conformations of A2062 may help CTY to improve drug activity and allosteric modulation.

The conformation rearrangement of A2062 may be related to the species-specific and macrolide-specific binding, and the position and interactions of the water molecule nearby may also depend on different complex structures and species, which may form bridge and stabilize the interactions between macrolide and NPET. The water molecule bound with the swayed A2062 may also interact with the translating peptide. The early studies have shown that m^2^A2503 may involve possible *syn-anti-*conformation transition when the specific nascent peptide is present in the NPET, and mutations of residues A2062/A2503 prevent stalling [40,41]**.** Our structure implies that the dynamic A2062 may also involve conformation transition during CTY binding, and the water molecule bound to A2062 might involve the translation control by allosteric modulation.

With significant alternative conformations found in *Mtb* ribosome-CTY complex in this study, the alternative conformations with minor population are identified from ribosome structures in complexes with SOL and TEL. Besides the species-specific feature, A2062 dynamics exhibits a specific response to some macrolides with certain chemical groups, such as 6-methoxy and alkyl-aryl side chain. A2062 may prefer the alternative conformations when the methoxy is presented at the binding interface like CTY, while the alkyl-aryl side chain of macrolide tends to reduce the distribution of dynamic rotation, such as SOL and TEL [43]. As the allosteric modulation plays an important role in translation control, and A2062 dynamics induced by CTY binding may provide key contribution to its superior potency without increasing binding affinity compared to ERY, according to the specific Mtb ribosome in this study and recent reports [43].

Besides the potential to break the N6-methoxy Bridge for *syn-*A2062, mutants A2062G/A2062U for the swayed A2062 may alter its dynamics and reduce binding affinity by disrupting the interactions with water molecule, neighbour bases and NPC, which is also consistent with CTY being reported as the most potent drug among many methylation derivatives of ERY as well as the drug resistance caused by mutants A2062G/U [8,9,39–41].

Remarkably, the recent report that a water molecule with H-bonds between A2058 and the desosamine dimethylamino is critical for ERY resistance caused by dimethylation of A2058, but the mechanism for streptogramin B remains enigmatic [34]. To date, the corresponding water molecule near A2058 could neither be identified by the map density from our structures nor the 2.4 Å cryo-EM 70S ribosome structure from *Sau* [24]. Therefore, the specific role of the water molecules near the ribosome-antibiotics interface is still not fully understood, which needs further studies on high-resolution structures of various species during different translation processes[34,40,41].

Interestingly, the methyl groups of desosamine moiety may contact multiple charged atoms from A2503, G2505 and A2059 within 3–4 Å distance, together with the possible water molecules and ions in the surrounding solution (Figure 4, S2). The significant effects of collectively weak interactions, like C–H H-bonds and π interactions, may play an indispensable role in binding affinity and specificity, which could help to reveal the previously overlooked interactions and unknown mechanisms related to drug resistance (Table S2 and Figure 4, S2).

The cryo-EM technique is a powerful tool to solve ribosome-antibiotics structures, but the sample preparation of complexes, in most cases, requires small molecules with high water solubility and high affinity. Unfortunately, large portions of chemical drugs yield limited water solubility or limited affinity, which is an obstacle for solving high-resolution cryo-EM structure.

With similar ideas used in the crystal soaking with antibiotics, the antibiotics binding reaction was performed for the cryo-EM samples by increasing the CTY concentration for binding and then reducing the concentration of organic reagent after the reaction. Therefore, our sample preparation methods provide an initial effort in drug discovery to overcome the challenge of drug insolubility or affinity limitation.

CTY is currently investigated for possible application in synergistic/combination anti-TB drug treatment for MDR/XDR pulmonary TB. LZD is another important anti-TB oxazolidinones targeting the 50S ribosomal subunit, and it would be important to address the specificity of its binding to *Mtb* ribosome when compared with CTY. CTY together with water molecules and specific NPC allosterically affect the conformation of U2585 in PTC where LZD also directly contacts with U2585, which may imply the synergistic effect of CTY in anti-TB therapy. The allosteric dynamics of A2062 is important to regulate the protein translation, which may have a great potential to improve the drug efficacy towards the dynamic modulation in translational control. The new insight gained from macrolide specific dynamics of A2062 by ribosome-CTY complex in this study, may help to rationally design the next generation anti-TB drugs to fight against MDR-TB.

## Supplementary Material

Supplemental Material
