# Supplementary material for: Cryo-EM structure of Mycobacterium tuberculosis 50S ribosomal subunit bound with clarithromycin reveals dynamic and specific interactions with macrolides
Source: Emerg Microbes Infect. 2022 Jan 21;11(1):293–305. doi: 10.1080/22221751.2021.2022439 (PMC8786254; doi:10.1080/22221751.2021.2022439)
Supplement: Supplemental Material [file TEMI_A_2022439_SM4688.docx]

Cryo-EM structure of *Mycobacterium tuberculosis* 50S ribosomal subunit bound with clarithromycin reveals the dynamic and specific interactions with macrolides

**Supplementary materials**

**Table S1. The *Eco* numbering of key nucleotides of *Mtb* 23S rRNA**

| **Nt** | ***Mtb*** | ***Eco*** | **Mutants** |
| --- | --- | --- | --- |
| A | 2296 | 2058 | G/U/C |
| A | 2297 | 2059 | C/G |
| G | 2299 | 2061 |  |
| A | 2300 | 2062 | G/U |
| A | 2741 | 2503 |  |
| G | 2743 | 2505 |  |
| C | 2848 | 2610 |  |
| U | 2849 | 2611 |  |

*Note: T thermophiles* (*Tth*), *D radiodurans* (*Dra*), *E coli* (*Eco*), *S aureus* (*Sau*), *H marismortui* (*Hma*), Nt: Nucleotide

**Table S2 The binding affinity between CTY and *Mtb* ribosome**

| **Ligand Receptor Interaction Distance(Å) E(kcal/mol)** |
| --- |

Dimethylamino (C28) O5'(G2505) H-donor 3.86 -0.7

Dimethylamino (C28) O4'(G2505) H-donor 3.12 -1.4

Dimethylamino (C29) N1 (A2059) H-donor 3.25 -1.8

Dimethylamino (C29) O2'(A2503) H-donor 3.45 -0.5

Dimethylamino (C29) OP2(G2505) H-donor 4.09 -0.9

2’-OH (O8) N1 (A2058) H-donor 2.93 -3.2

Note: the total binding affinity contributed by C-H H-bonds is about -5.3 kcal/mol, which is more significant than the standard H-bonds between 2’-OH of desasomine and N1 of A2058.


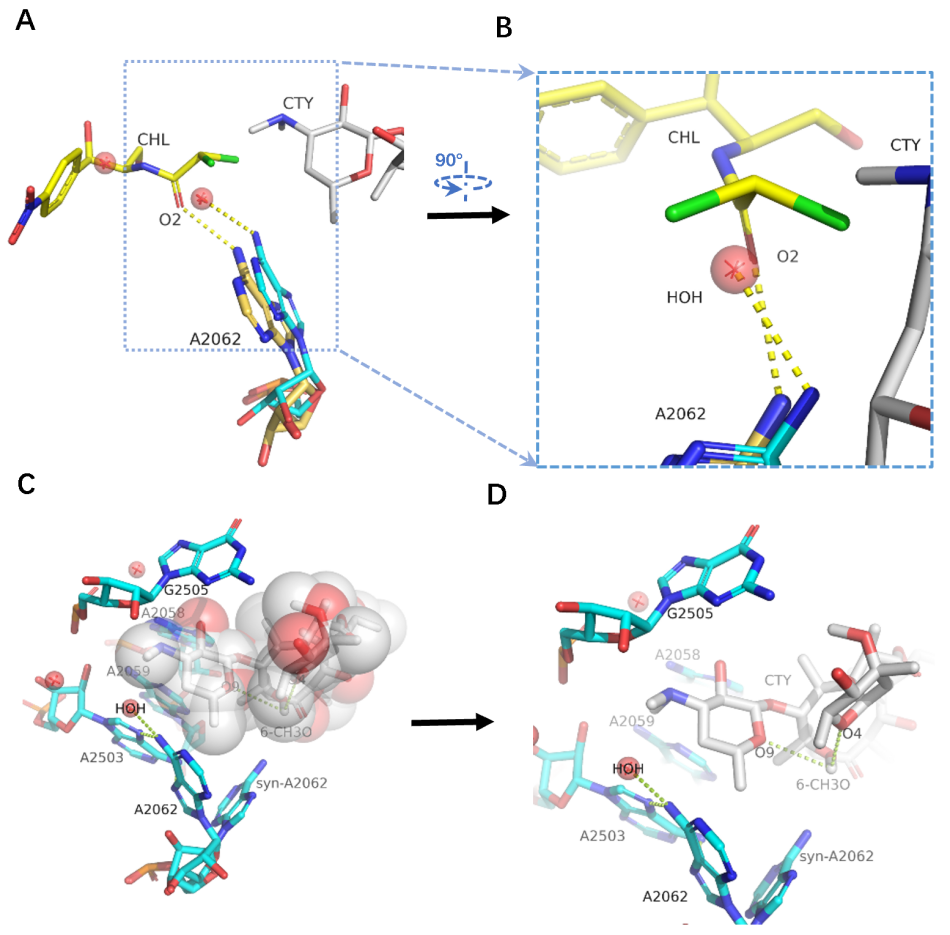


Fig. S1. The water molecule at the interface between the swayed A2062 and desosamine of CTY. A) the almost identical position for the water molecule and the dichloroacetic moiety of CHL (water molecule to O2 of the dichloroacetic distance: 1.1Å, or 0.3 Å if further applied base alignment of A2062); B) closeup view after 90 degrees rotation; C) the vdW contacts of CTY with the nucleotides in NPET; D) closeup view without spheres. Notes: the distance of water molecule to N6 of rotated A2062 is 2.7Å in CTY-Mtb complex, and O2 of the dichloroacetic to N6 of rotated A2062 is 3.0Å in CHL-*Tth* ribosome complex.


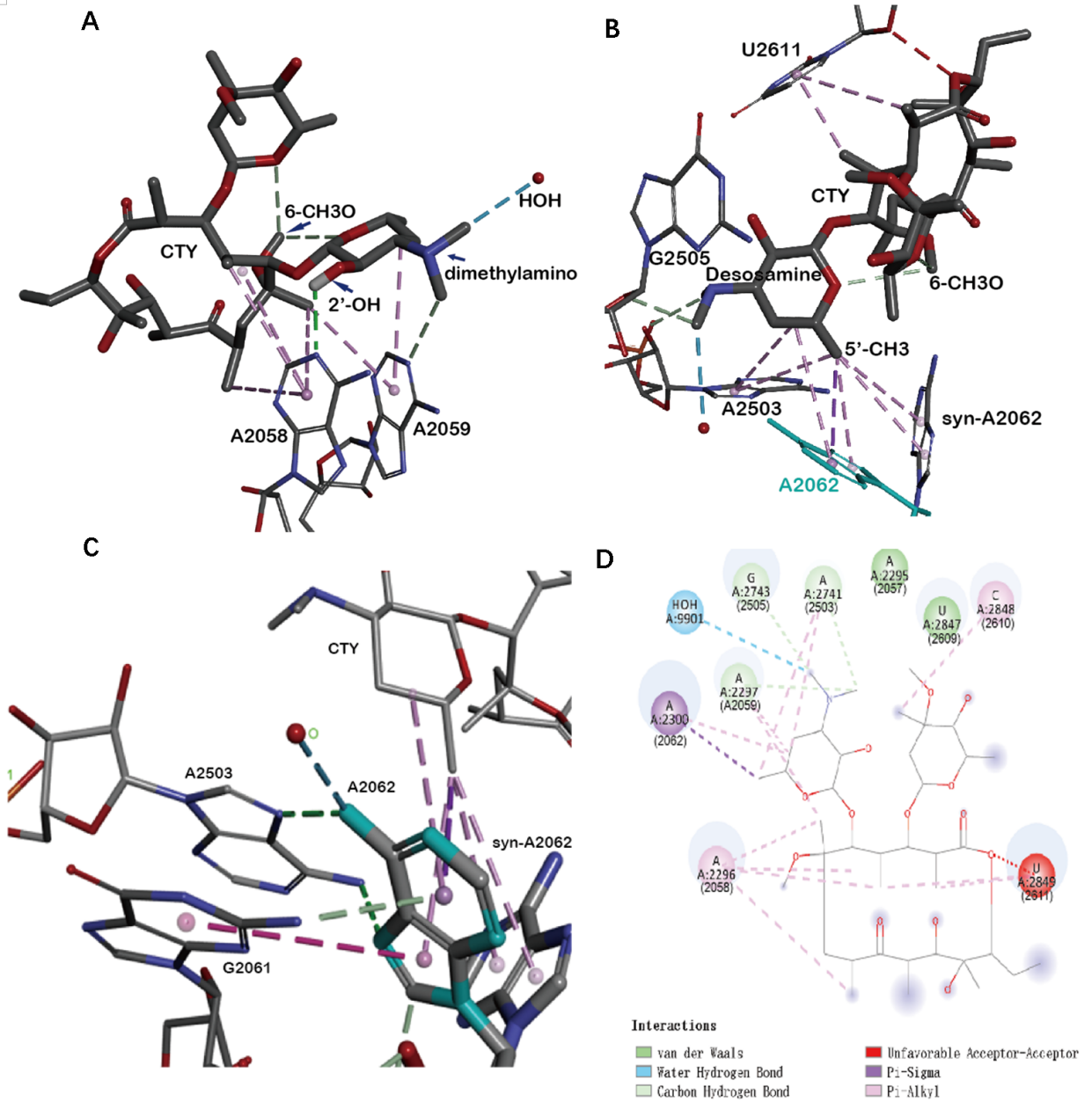


Fig. S2. The additional π interactions between CTY and nucleotides A2058, A2059, A2062, m^2^A2503, U2611 in NPET, together with the interaction 2D map. A) interactions of A2058 and A2059 with CTY; B) interactions of A2062, m^2^A2503 and U2611 with CTY; C) interactions of A2062 with CTY, G2061 and m^2^A2503; D) the 2D plot of interactions between CTY and ribosome. Note that the lactone ring of CTY near the unfavorable binding U2611 is corresponding to weak density in Fig 1B; Figures were generated by Discovery Studio with default settings.


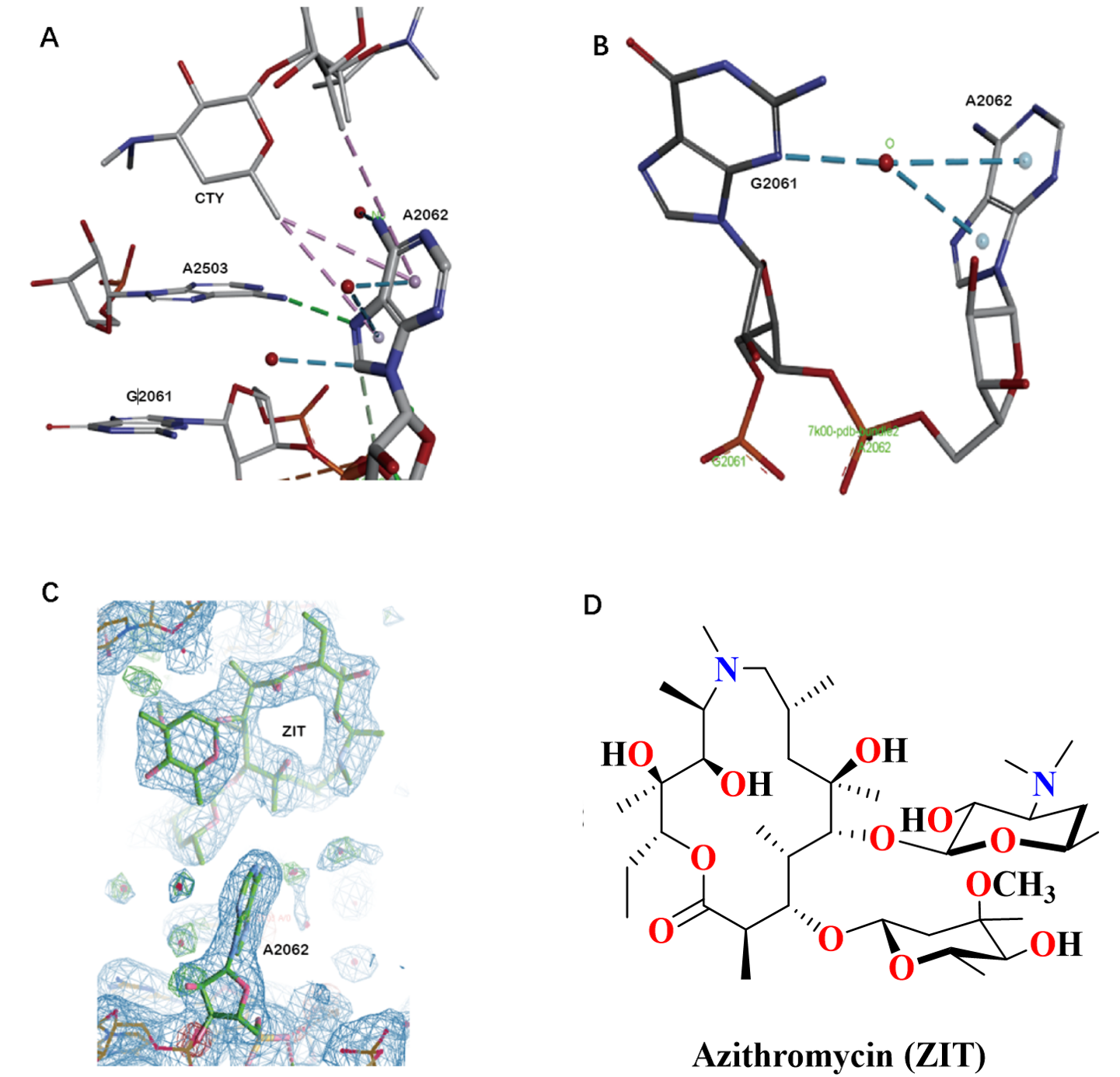


Fig. S3. The water molecules bound to the anti-A2062 (unrotated conformation) and contributing to its conformation stabilization. A) interactions of A2062 with the water molecules, CTY and m^2^A2503; B) the interaction mediated by the conserved water molecule between A2061 and A2062; C) the 2.4Å density map of unrotated A2062 from ribosome structure (PDB: 1YHQ, other two water molecules near A2062 were not shown); D) chemical structure of azithromycin (ZIT). Note: Figures were generated by Discovery Studio with default settings, and the interactions were shown with the same indications shown in above Fig.S2.


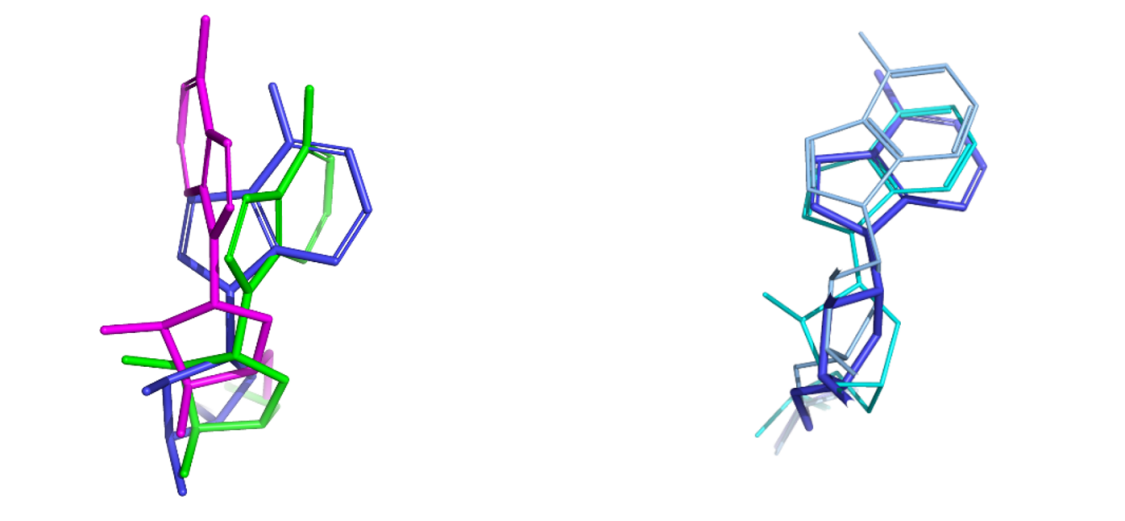


(A) *Mtb* (this work), *Msm* (5o61), *Mtb* (5v7q); (B) *Msm* (5o61), *Msm* (5xym), *Msm* (5zeb),

Fig. S4. The A2062 conformation in the vacant ribosome structure.

1. *Mtb*: Green; 5o61: deep blue (*Msm*); 5v7q: pink (*Mtb* apo ribosome 3.7A); B) 5xym: cyan (*Msm*); 5zeb: light blue (*Msm; note that* density map is weak due to flexible structure of A2062)


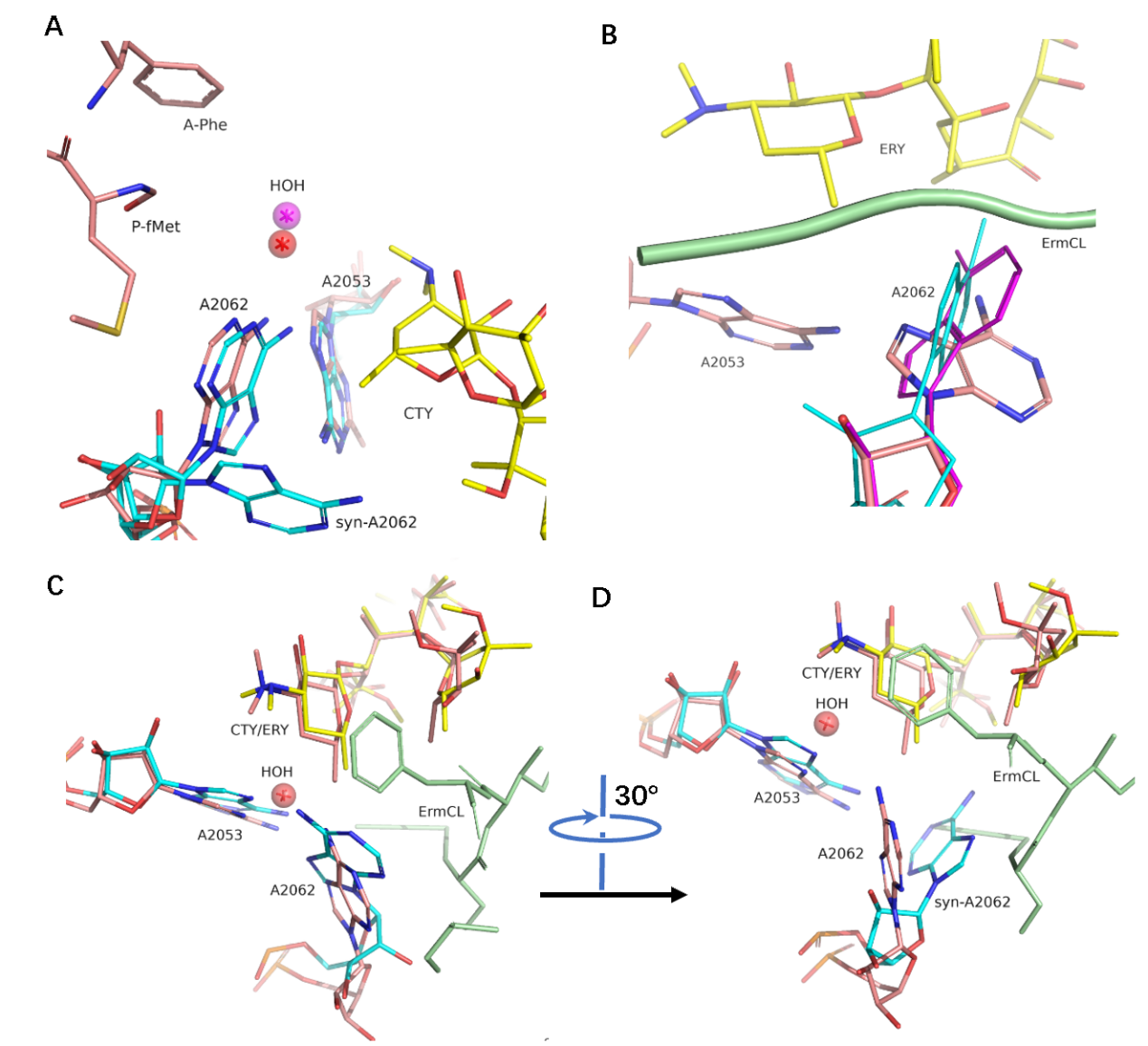


Fig. S5. The possible interactions of A2062 and the bound water molecule with the NPC or ErmCL within NPET (interaction analysis based on structure alignment). A) the interactions of the swayed A2062 and the bound water molecule (red) with the aminoacylated tRNAs at A-site and P-site (carbon in orange, water molecule in pink: 6XHV), carbon in cyan for Mtb ribosome, and carbon in yellow for CTY ; B) the interactions of ErmCL peptide with the rotated A2062 (without swayed, carbon in orange, PDB code 3J5L) comparing to the ribosome structure from *Mtb* (non-swayed A2062 in cyan) and *Eco* (in pink, PDB code 4V7U); C)the interactions of ErmCL peptide (green) with the swayed A2062 (carbon in orange, PDB code 3J7Z) and water molecule (carbon in cyan for *Mtb* ribosome with the swayed A2062, CTY in yellow); D) the possible structure confliction of ErmCL peptide(green) with the non-swayed A2062 from *Mtb* ribosome(carbon in cyan); Notes: C) to D): 30 degree rotation**;** *Mtb* ribosome: carbon in cyan by default;


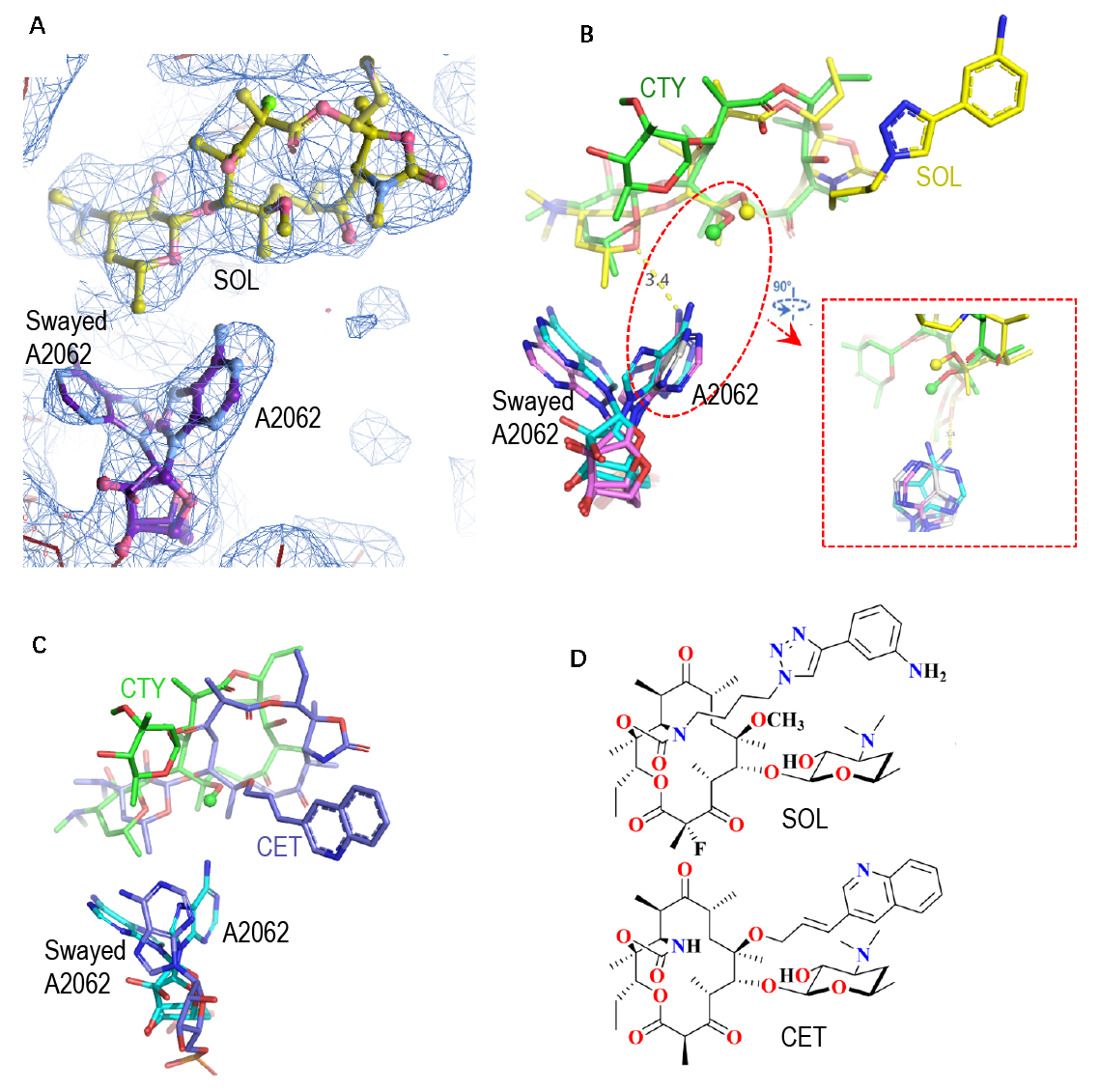


Fig. S6. The conformation changes of A2062 binding to the macrolide SOL and CET (with different substitution at 6-OH of lactone ring). A) The alternative conformation of A2062 interacting with SOL (PDB code 4WWW, the weak density of swayed conformation indicating the minor population of conformation) ; note: visible density contoured at 0.3~0.8σ**.** B) structure comparison of A2062 and macrolide between ribosome-SOL and Mtb ribosome-CTY complexes (the carbon for ribosome-SOL complex/ PDB code 4WWW: SOL in yellow/A2062 in pink; ribosome-CTY complex: CTY: in green/A2062 cyan; A2062 in gray for Eco ribosome-ERY/PDB code 4V7U; 6-Methoxy in small spheres); C) the structure of ribosome-CET comparing to the ribosome-CTY structure(carbon in deep blue for ribosome-CET complex/PDB code 1NWX; no density was available for this structure, the further analysis was ignored); D) the chemical structures of CET and SOL. Note: the interface differences between A2062 and macrolide are shown in the insert of B) and C);


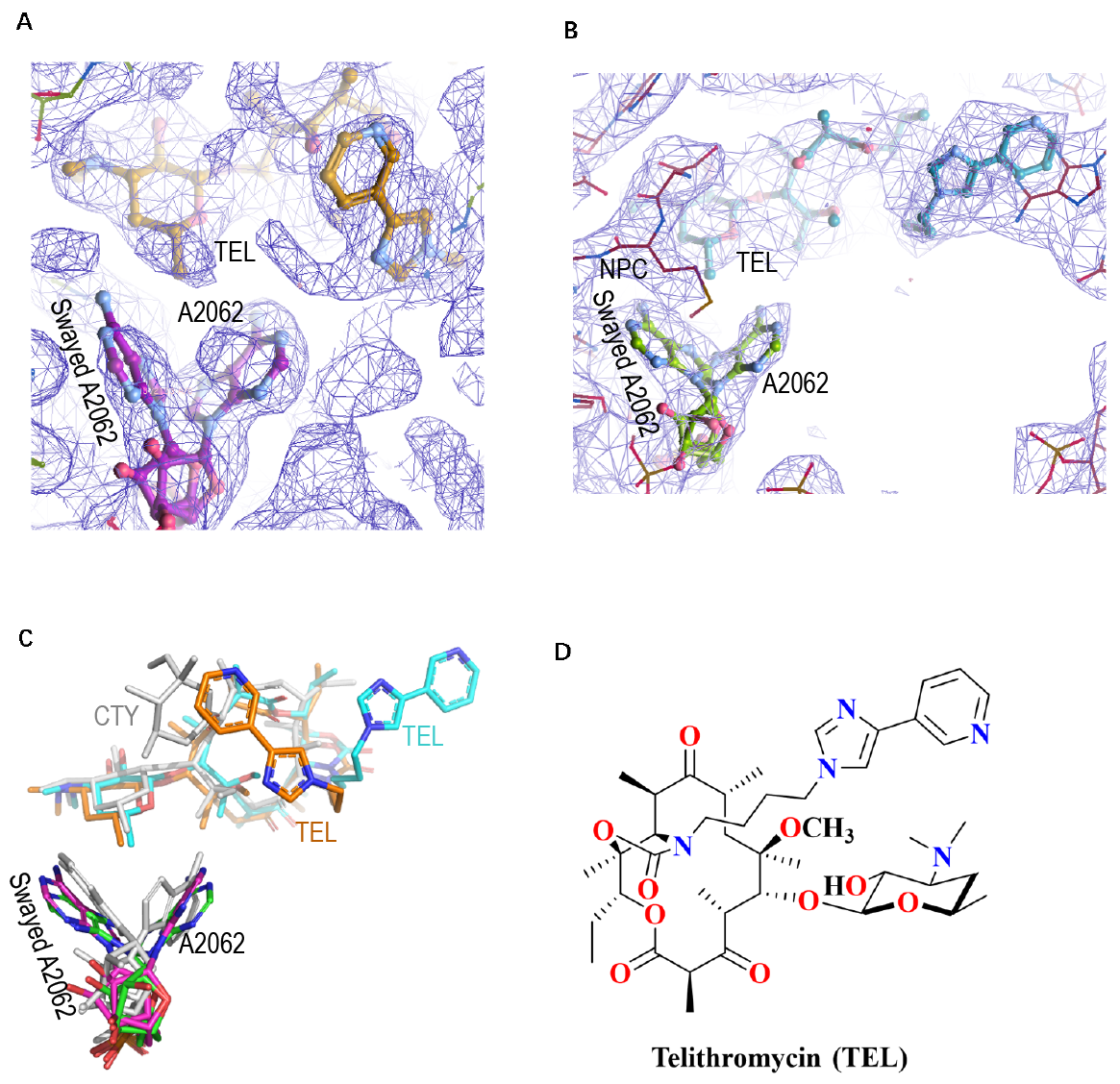


Fig. S7. The alternative conformation identified from ribosome-TEL complexes. A) minor rotation conformation identified in *Hma* ribosome-TEL complex (PDB code: 1YIJ); note: visible density contoured at 0.3~0.8σ. B) minor unrotated conformation identified in *Eco* ribosome-TEL-NPC complex (PDB code: 7NSQ); map contoured at 0.8σ. C) conformation comparison from different complexes (carbon in grey for ribosome-CTY complex; A2062 in green for 7NSQ; A2062 in pink for 1YIJ); D) chemical structure of TEL.
